# Solitary Contralateral Adrenal Metastases after Nephrectomy for Renal Cell Carcinoma

**DOI:** 10.1100/tsw.2004.39

**Published:** 2004-08-31

**Authors:** Nikolaos Antoniou, Demetrios Karanastasis

**Affiliations:** General Hospital of Athens, Athens, Greece

## Abstract

Two cases are reported of patients with a single metastasis in the contralateral adrenal, one and nine years respectively after nephrectomy for localized cancer in the opposite kidney. These types of metastases are usually asymptomatic they do not appear with signs of adrenal insufficiency, they are detected incidentally and the diagnosis is confirmed mainly with CT scan, which comprises the method of choice for the detection of such types of metastases. Many adrenal metastases probably have been overlooked in the past when advanced imaging techniques were not available. Both patients underwent adrenalectomy and replacement therapy with corticosteroids. Approximately 50% of all patients with renal cell carcinoma either present with metastases at diagnosis or will have metastatic disease after nephrectomy1. In order of decreasing frequency, the most common metastatic sites are the lungs, lymph nodes, liver, bone, contralateral kidney and ipsilateral adrenal glands. Adrenal involvement has been reported in 7 to 19% of autopsystudies. Solitary metachronous metastatic involvement of the contralateral adrenal from renal cell carcinoma is rarely diagnosed during life and only 4 cases have been reported. Recent advances in imaging techniques have allowed the diagnosis of adrenal involvement by renal cancer. Two cases of contralateral adrenal metastasis are reported 1 and 9 years after radical nephrectomy for localized renal cell carcinoma. Both patients underwent adrenalectomy and steroid replacement therapy.

## CASE 1

Female, aged 68 years, underwent radical right nephrectomy for localized renal cancer 1 year ago (Fig. 1). During surgery the existing embolus in the right renal vein was removed. Distant metastases were not found. During a follow up examination 6 months later, the CT-scan showed a suspicious enlargement of the left adrenal (Fig. 2). The ultrasound tomography was not helpful in identifying the nature of the enlargement. MRI (Fig. 3) and selective angiography (Fig. 4) showed either a suspicious primary tumour of the left adrenal or the presence of a metastatic disease. The biochemical and hormonal laboratory tests were normal. A left adrenalectomy was performed through a left thoracoabdominal incision and a tumour (4 x 1 cm in diameter) was removed (Fig. 5). The pathological examination of the tumour revealed a

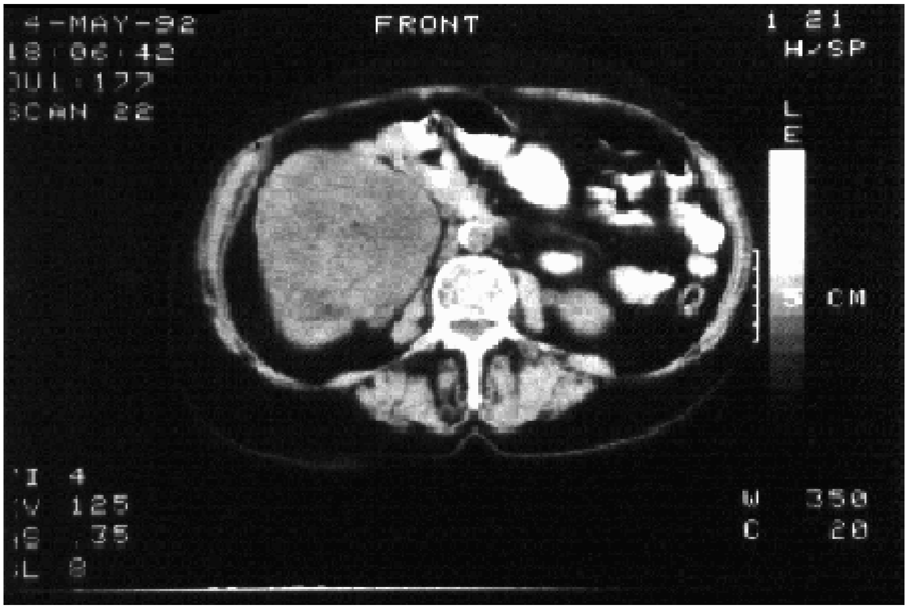

**FIGURE 1**. Case 1. CT Scan. Renal cancer of the right kidney.

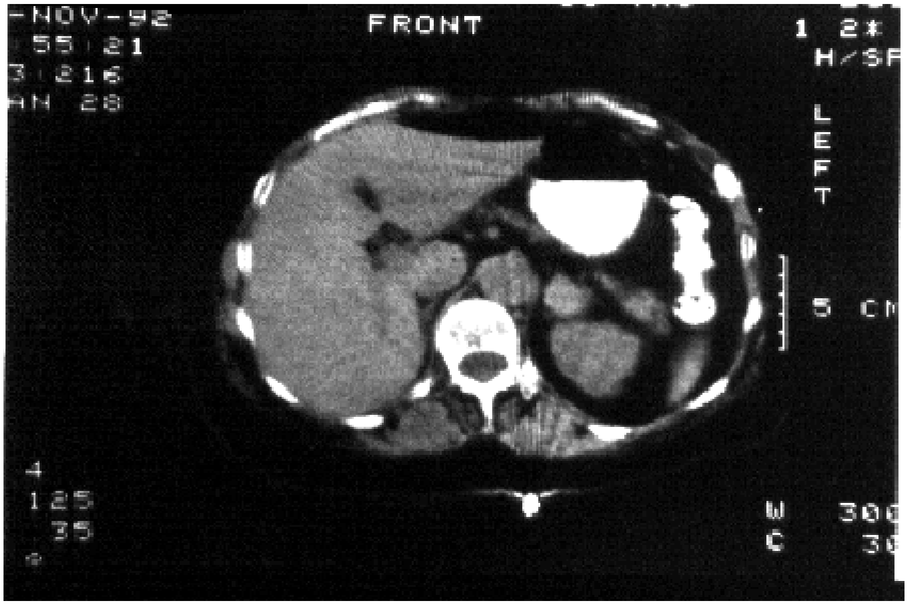

**FIGURE 2.** Case 1. Abdominal CT scan of the left adrenal mass.

metastatic renal tumour of moderate differentiation and of the same type of cancer which was removed before one year (Fig. 6). Preoperatively the patient started replacement therapy with hydrocortisone 100 mg tid until the 3rd postoperative day, followed by cortisone acetate 25 mg per os for life. She is in good condition 4 months after surgery.

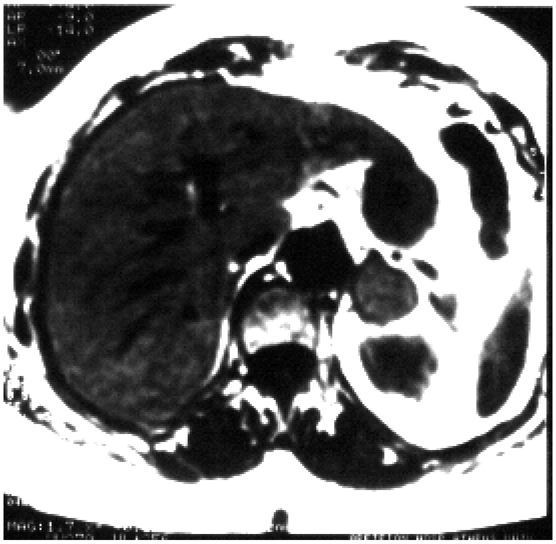

**FIGURE 3**. Case 1. Magnetic Resonance Imaging of the abdomen of the suspicious left suprarenal mass.

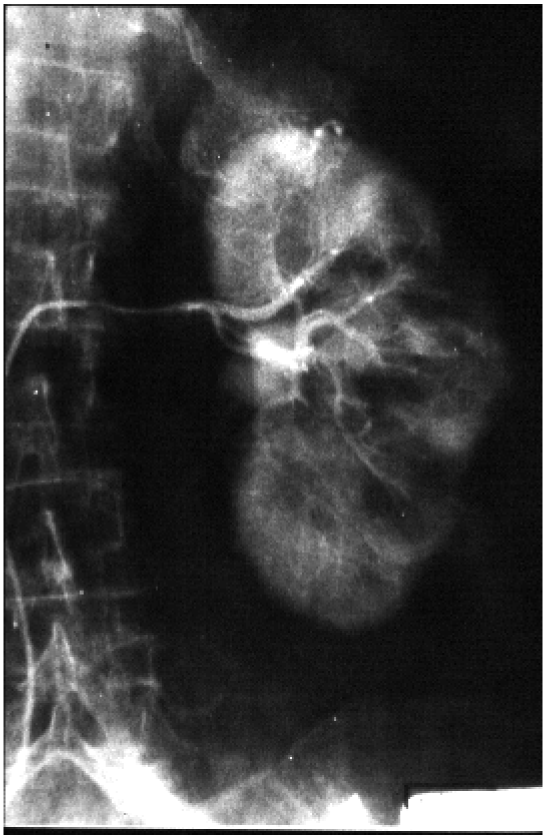

**FIGURE 4.** Case 1. Left Renal arteriogram during arterial phase: Left lower adrenal artery supplies faintly vascular mass above left kidney.

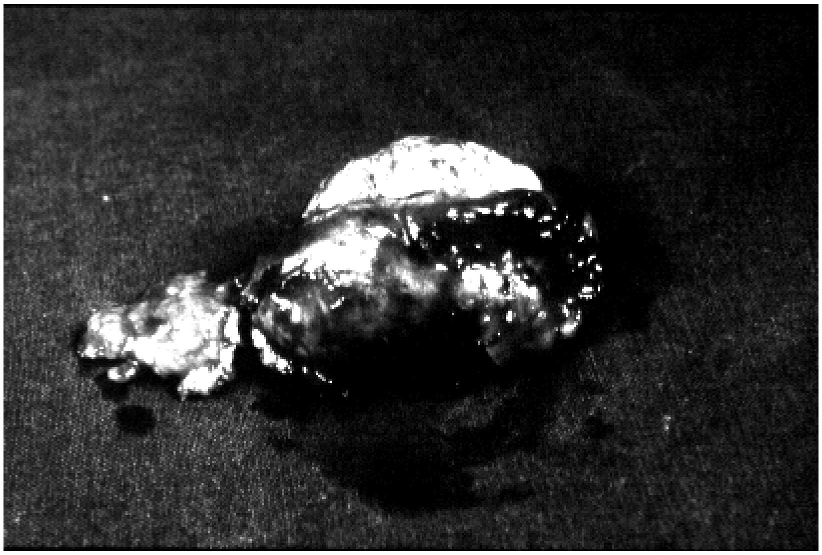

**FIGURE 5.** Case 1. Resected left adrenal (4 x 2 x 2 cm.).

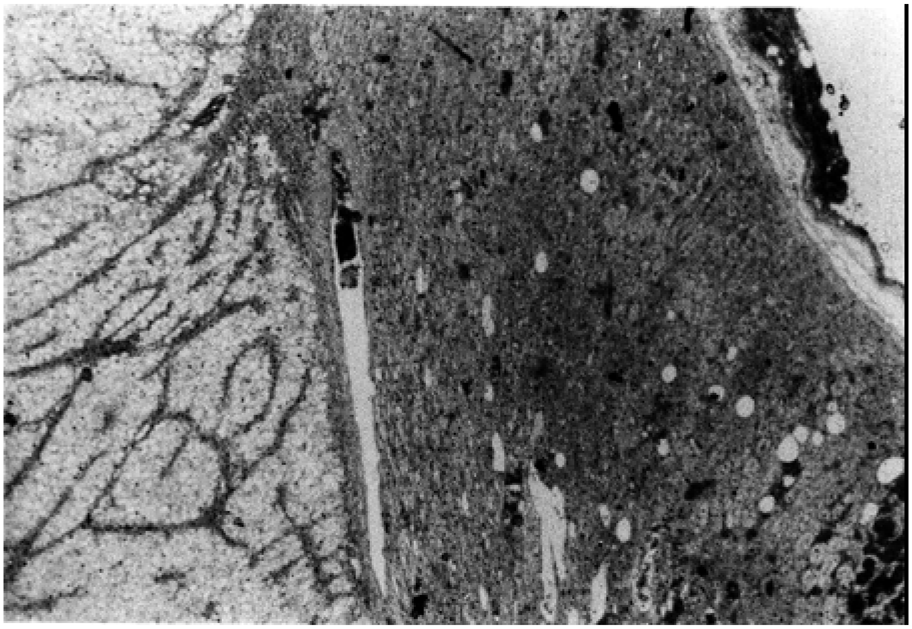

**FIGURE 6.** Case 1. Histological section of the adrenal metastasis from renal cell carcinoma (H + E x 100).

## CASE 2

Male, aged 72 years, was admitted due to fever, anemia and loss of bodyweight - approximately 7 kg. in the previous 2 months. In 1981 he underwent a left radical nephrectomy due to cancer of the corresponding kidney. After being admitted, only anemia (Hematocrit = 29.8) and a rise in ESR (9Omm) were discovered. The intravenous pyelography revealed a mass in the upper pole of the right kidney and the CT-scan confirmed its presence in the area of the right adrenal (Fig. 7). Selective angiography was then performed, which showed the presence of a hypervascular mass (Fig. 8). Further tests with bone scans and CT-scans were negative for the presence of metastases. The biochemical and hormonal

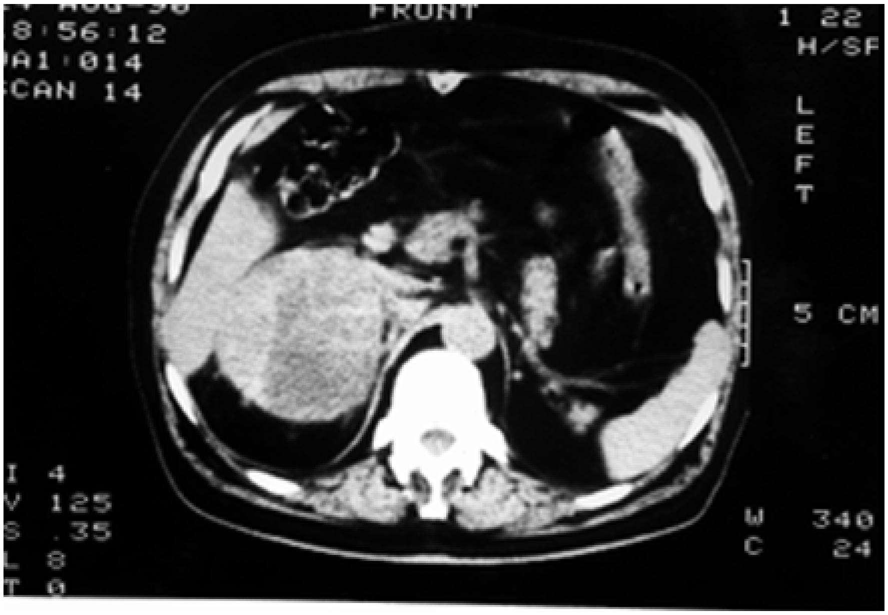

**FIGURE 7.** Case 2. Abdominal CT scan of the right suprarenal mass.

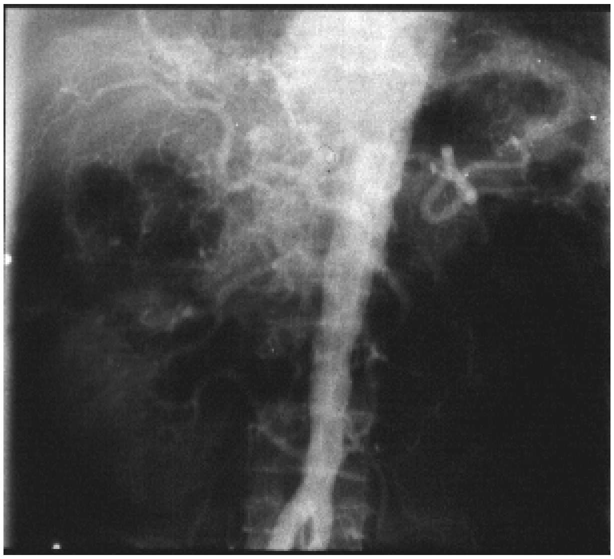

**FIGURE 8.** Case 2. Selective right renal arteriogram with highly vascular suprarenal mass which displaces kidney interiorly.

laboratory tests were normal. The tumour was removed en-bloc through a right thoracoabdominal incision. The pathological examination showed a mass of 10 cm. (Fig. 9) which histologically presented the characteristics of a metastatic renal cancer of moderate differentiation and of the same type of cancer of the contralateral kidney removed 9 years ago (Fig. 10).

The patient immediately started replacement therapy with corticosteroids he was well, until 7 months later when the CT-scan showed a suspicious mass between right kidney and inferior vena cava (Fig. 11). Immunotherapy was started with Interferon a-2a of 18 MU IM 3 times a week for 3 months but with no response. Generalized metastases developed and he died nine months later.

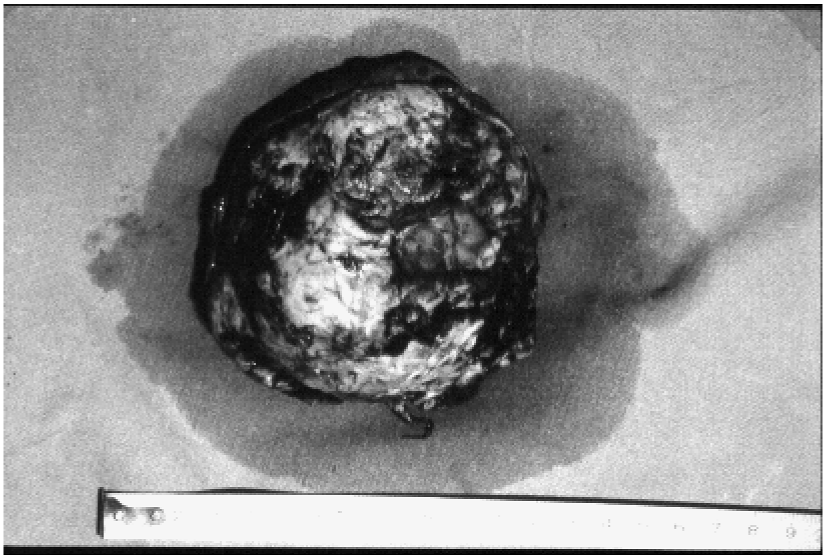

**FIGURE 9.** Case 2. Gross resected right adrenal (10 x 8 x 4 cm.).

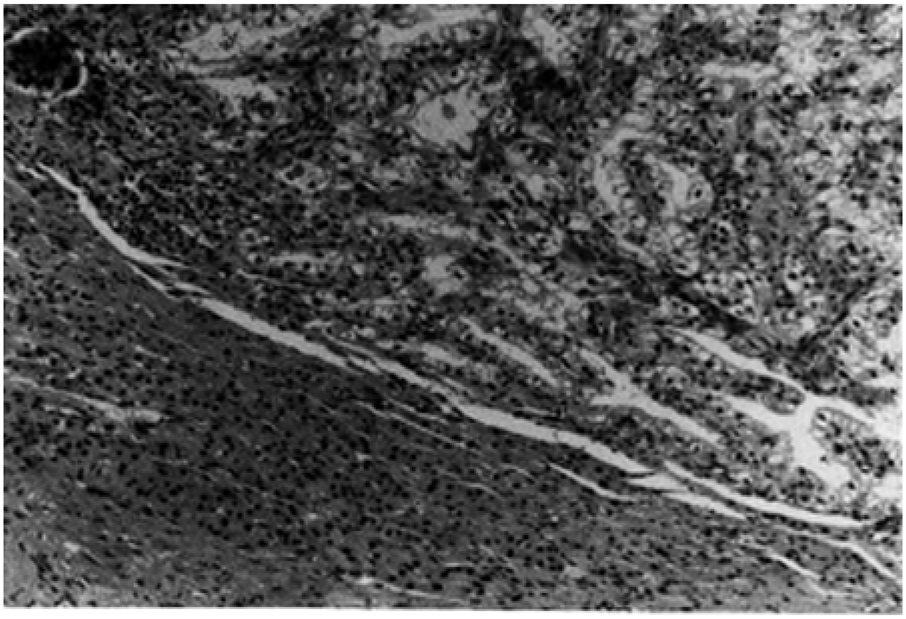

**FIGURE 10**. Case 2. Histological appearance of the adrenal tumor.

## DISCUSSION

Adrenal metastases usually result from breast cancer, lung cancer, and melanoma[6]. Approximately, 3 percent of all adrenal metastases are from renal cancer. The incidence of the localization in the adrenalsis probably explained by the type of adrenal blood supply, by their high flow volume and by the type of their capillary network, which has a sinusoidal vascular pattern. On the contrary, the spleen-which has the same type of blood supply is a rare site for metastases.[7]

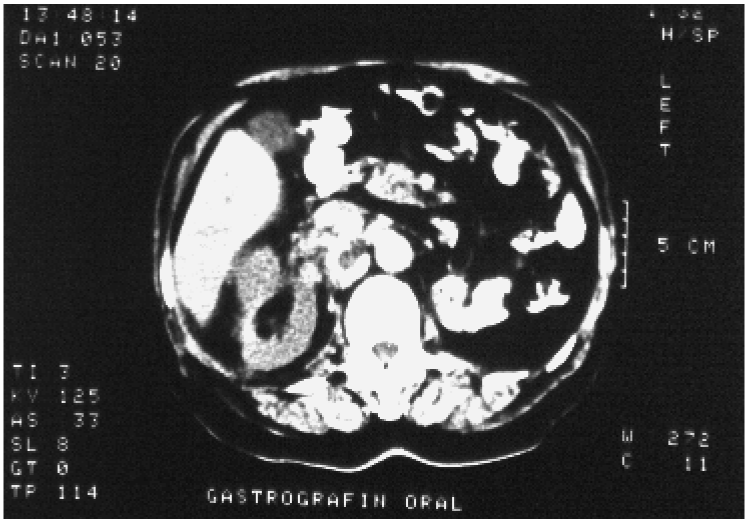

**FIGURE 11.** Case 2. The suspicious mass between right kidney and IVC.

Solitary metastases in the adrenals have been reported in 1.6-3.6% of patients with renal cancer[8]. The most common sites for metastases of the renal cancer is the lung and the bones[2]. Contralateral metastases from renal cancer are very rare. Metachronous metastatic involvement of the contralateral adrenal from renal cell carcinoma is rarely diagnosed during life, and with only 4 cases mentioned in the literature. [4,5]

**TABLE 1**
**Renal Cell Carcinoma with Metachronous Solitary Adrenal Metastases**

| Reference | Age / Sex | Side of Primary | Detection of Adrenal Metastases | Diagnostic Procedure | Treatment | Follow-up |
|---|---|---|---|---|---|---|
| T.K. Huisman and J.P.Sand | 46/M | R | 2 years after nephrectomy | CT | Adrenalectomy (L) | +7Mo NED died +20mo |
| JR (2 cases) | 69/M | R | 4 years after nephrectomy | CT | Adrenalectomy (L) | +12Mo NED sol. lung met. Excised alive +36 mo |
| J. Plawner | 70/M | L | 6 years after nephrectomy | CT | Adrenalectomy (R) | +60Mo NED |
| (2 cases) | 57/M | R | 7 years after nephrectomy | CT | Adrenalectomy (R) | +32Mo NED |

R = Right
K = Left
CT = Computerized Tomography
NED = No Evidence of Disease

A characteristic of adrenal metastases is that they either remain "silent", without showing any symptoms, (as in Case 1) or they are diagnosed when they become very large (as in Case 2). The silent nature of such metastases is explained as is known that even one tenth of the remaining adrenal tissue is capable of regulating all normal body functions without the appearance of adrenal insufficiency and with normal cortisone and adrenaline values in the blood[9].

There is currently general agreement that the CT scan of the thorax and of the abdomen is the more precise method for the diagnosis of metastases after nephrectomy for renal cancer and that the IVP and chest X-ray have been replaced by CT scan.

This is also true for the diagnosis of adrenal metastases where the chief method of detection is CT scan. The appearance of these tumours have characteristics similar to those of solid renal tumours.

MRI probably gives just as good information. Angiography is still helpful in the diagnosis of these localizations since the lower adrenal arteries are usually well demonstrated, especially on the right. The left adrenal artery is less often visualized[12].

Low-grade, low-stage renal cell carcinoma carries a good prognosis[10]. It is therefore desirable to diagnose metastatic lesions while they are solitary and small. Early diagnosis facilitates aggressive surgical resection. The management of such cases is always adrenalectomy and replacement therapy with corticosteroids for life.

In conclusion, contralateral solitary metastases in the adrenals, after nephrectomy for renal cancer, although rare, are asymptomatic. They do not appear with signs of adrenal insufficiency. They are detected "incidentally" mostly with CT-scan during the follow-up of patients with renal cancer.

A close follow-up of all patients who have undergone nephrectomy for renal cancer is necessary, especially for patients who are asymptomatic for many years after the primary operation. Many adrenal metastases probably have been overlooked in the past when advanced imaging techniques were not available.
